# Electrostatic Field Invisibility Cloak

**DOI:** 10.1038/srep16416

**Published:** 2015-11-10

**Authors:** Chuwen Lan, Yuping Yang, Zhaoxin Geng, Bo Li, Ji Zhou

**Affiliations:** 1 State Key Laboratory of New Ceramics and Fine Processing, School of Materials Science and Engineering, Tsinghua University, Beijing 100084, China; 2 Advanced Materials Institute, Shenzhen Graduate School, Tsinghua University, Shenzhen, China; 3 School of Science, Minzu University of China, Beijing, 100081, China; 4 School of Information Engineering, Minzu University of China, Beijing, 100081, China

## Abstract

The invisibility cloak has been drawing much attention due to its new concept for manipulating many physical fields, from oscillating wave fields (electromagnetic, acoustic and elastic) to static magnetic fields, dc electric fields, and diffusive fields. Here, an electrostatic field invisibility cloak has been theoretically investigated and experimentally demonstrated to perfectly hide two dimensional objects without disturbing their external electrostatic fields. The desired cloaking effect has been achieved via both cancelling technology and transformation optics (TO). This study demonstrates a novel way for manipulating electrostatic fields, which shows promise for a wide range of potential applications.

The invisibility cloak refers to a device with the capability of shielding the object from sight without disturbing its external physical field. The first successful research on invisibility cloak was based on theoretical prediction and experimental demonstration^1^,^2^. Since then, this intriguing concept has aroused increasing interest, particularly in the field of manipulating electromagnetic waves^1^,^2^,^3^,^4^,^5^,^6^,^7^,^8^. Motivated by the fruitful achievements in electromagnetic waves, considerable progress has been made in studying other waves, including mechanical waves^9^, elastic waves^10^, and matter waves^11^.

In recent years the concept of invisibility has also been extended to magnetic fields^12^,^13^, dc electric fields^14^,^15^, thermal fields^16^,^17^,^18^,^19^,^20^,^21^, mass diffusion^22^,^23^ and diffusive light scattering^24^. In addition, the concept for manipulating static fields has also been used to develop fruitful devices^25^,^26^,^27^,^28^,^29^,^30^. However, little attention has been focused on the manipulation of the electrostatic field in dielectric medium, except for theoretical studies on metamaterials^31^ and cloaking^32^. Since the electrostatic field is a wide-ranging concept in industry, agriculture and daily life, it is natural to expect that manipulation of the electrostatic field would find applications in various fields. For example, metals and high dielectric materials are usually used to shield or to detect the electrostatic field. However, their presence inevitably introduces disturbances to the surrounding field. Thus, it is intriguing and desirable to develop a cloak to cancel or reduce the disturbances.

In this paper, we report an electrostatic field invisibility cloak (EFIC) to shield an object without disturbing the external electrostatic field. Our contribution is twofold. Firstly, we have experimentally demonstrated an EFIC with a simple structure, which can be easily extended to micro-nanoscale and three-dimensional configurations. In addition, this work may be useful for further studies in electrostatics.

## Results

### Bilayer electrostatic cloak

**Figure 1:**
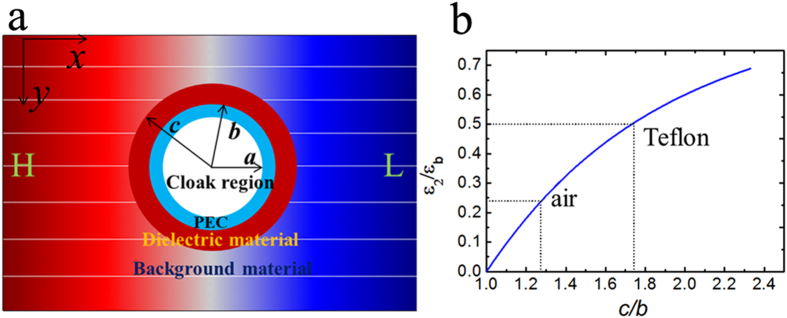
Design of electrostatic field invisibility cloak. (**a**) The physical model for EFIC. (**b**) The required relative dielectric constant for EFIC with radii ratio of the outer layer of cloak. The material candidates are marked by dash lines.

We start with EFIC based on the cancelling technology method. Figure 1a schematically illustrates the corresponding physical model where a uniform electric field ***E*** is produced. Assume that a two-dimensional object to be cloaked is placed in the field. Here, the idea of EFIC is to guide the electric field around the object without any distortion. Inspired by previous works, the bilayer structure can be easily designed to achieve this goal by directly solving the electrostatic field equation (see Methods). The required parameters are given by:


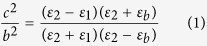


Here, *b* and *c* are the inner and outer radii of the coaxial cloaking tube. 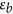, 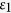 and 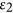 are the dielectric constants for the coaxial background material, inner layer and outer layer of the cloaking tube, respectively.

In our experiment, the inner layer of the cloaking tube is made of perfect electric conductor (PEC) or metal conductor, which in the static case has the dielectric constant 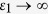. Note that this is a different approach from those in magnetic fields, dc electric fields and thermal fields, where *μ*, *σ, κ* of the inner layer are all set to be 0. The PEC or metal conductor is chosen due to its excellent electrostatic shielding performance. Furthermore, the metals are widely used in probes for electrostatic measurements. Since 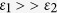, Equation 1 can be expressed as


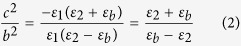


As a result, the required dielectric constant for the outer layer of the cloaking cylinder can be obtained:


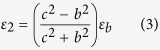


Figure 1b plots the dependence of the relative dielectric constant 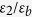 for the outer layer on the radii ratio 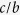. In our study, castor oil with the dielectric constant of 4.3 is used as the background medium. Figure 1b also gives the required geometry parameters when the outer layer is air 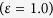 or Teflon 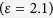, respectively. In our study, air is chosen as the outer layer, thus the required radii ratio 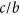 is 1.3. We choose a steel layer (SL) with 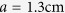, and 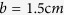 as the inner layer. Note that other good conductors can also be used. The geometry parameters for the air layer can be determined as: 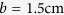, and 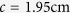. To verify the theoretical prediction, numerical simulations were carried out based on Multiphysics Comsol. Here three cases are discussed: a) background medium (castor oil); b) castor oil + steel layer (SL); c) castor oil + air layer (AL). In the simulations, the size of the modelling area is 15 × 15 cm^2^, and −1000 V potential is applied to two edges to generate uniform electrostatic field.

**Figure 2:**
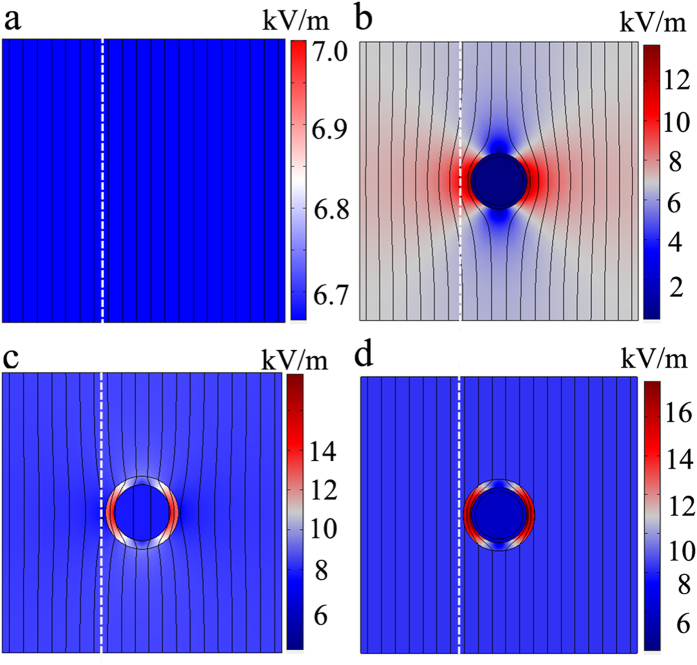
Numerical simulation. The simulation electric field and isopotential lines (black lines) for four cases: (**a**) castor oil. (**b**) castor oil +SL. (**c**) castor oil + AL. (**d**) bilayer cloak. The dotted white lines denote the measuring lines in the experiments.

Figure 2a–d gives the simulation results, where the electric field distribution and isopotential lines are plotted. Figure 2a–c, describe case a), case b) and case c), respectively, and Fig. 2d provides the results for the designed bilayer cloak. As seen in Fig. 2a, a uniform electric field and gradient potential can be generated. Figure 2b,c illustrate that the steel layer repels the isopotential lines and protects the interior from the external field, while air layer attracts the isopotential lines. For both cases, the isopotential lines and electric field are seriously distorted. In the case of the bilayer cloak, however, the electric field travels around the inner domain without any disturbance, as depicted in Fig. 2d. In contrast, the distortion for electric field and isopotential only occurs in air layer. Therefore, the inner domain is protected from the external field and thereby a perfect cloak is obtained.

### Experimental demonstration of bilayer cloak

**Figure 3:**
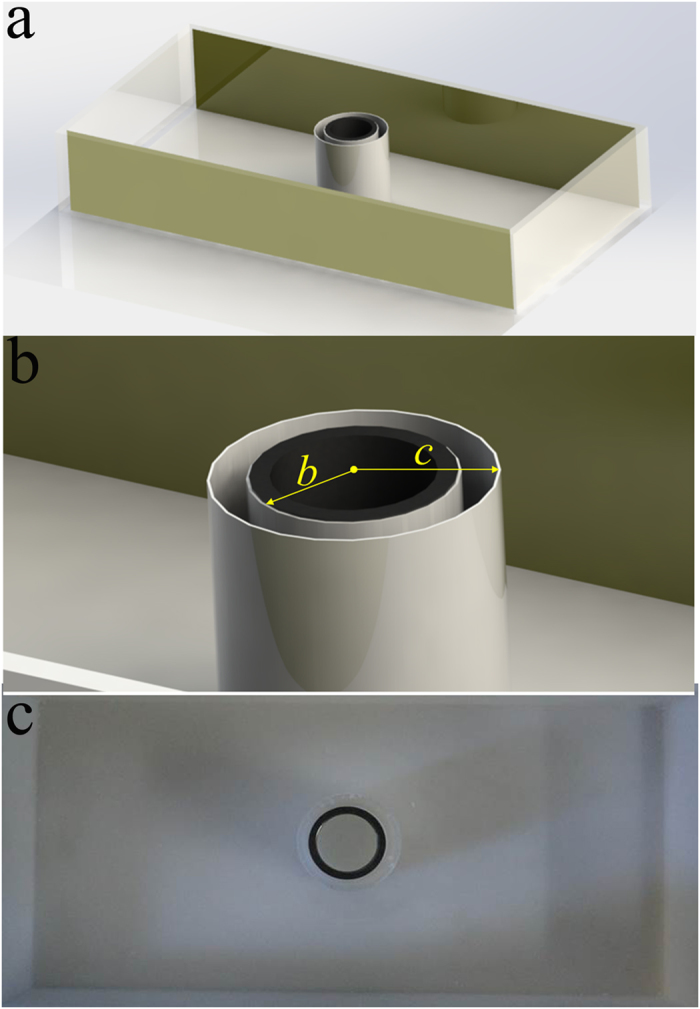
Experimental demonstration of electrostatic field invisibility cloak. (**a**) Schematic illustration for realization of EFIC (**b**) Schematic illustration of the fabricated sample. (**c**) Photograph of the fabricated sample.

This fabricated bilayer cloak shown in Fig. 3 is composed of commercially available steel tubing with following dimensions: inner radius *a* = 1.3 cm, outer radius *b* = 1.5 cm and height *h* = 5 cm, and it is placed in a photosensitive resin container. To construct the bilayer cloak described above, the steel shell tube is further wrapped by a photosensitive resin (ε = 2.0) shell container with the following dimensions: inner radius *b* =1.5 cm, outer radius *c* = 1.95 cm and height *h* = 5 cm. The photosensitive resin shell tube can be fabricated by SL process^33^. The thickness of the container tube wall is 0.45 mm. The shell container tube is filled with air, and a bilayer coaxial tube cloak is obtained. Simulations show the presence of the container tube produces very little influence on the performance.

**Figure 4:**
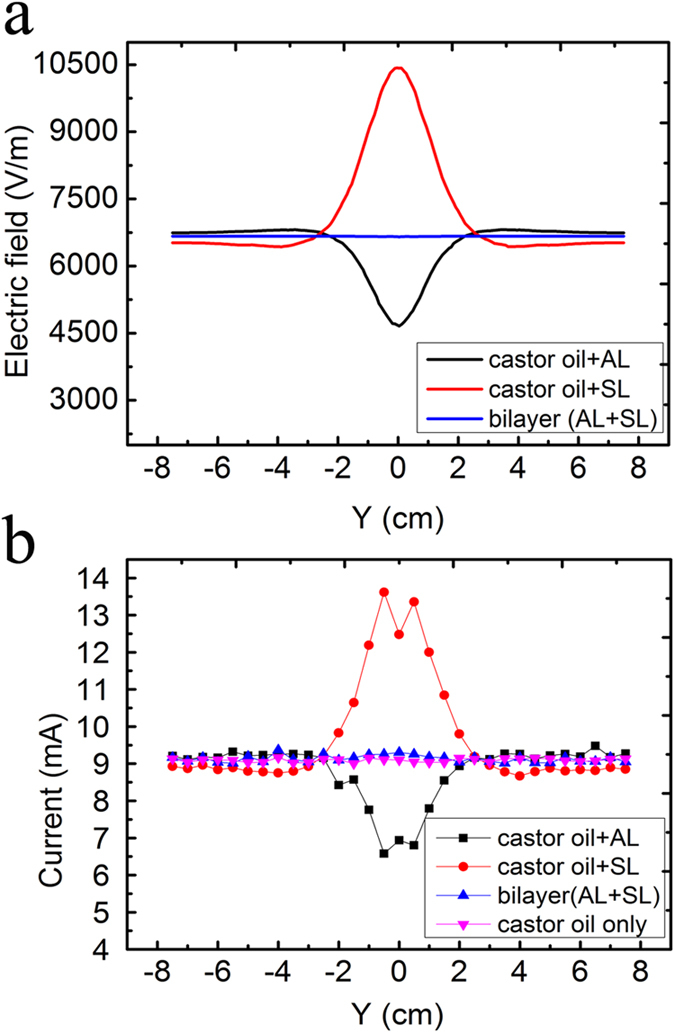
Characterization of electrostatic field invisibility cloak. (**a**) Simulated results of electric field for different cases. (**b**) The measured current in the electrostatic field measurement instrument for the corresponding cases, respectively.

In the experiment, the bigger container is filled with castor oil and two copper plates are used as electrodes. The electrodes are applied with −1000 V by electrostatic generator to create an electrostatic field in *x*-direction. The performance of cloak can be evaluated by measuring the electric field distribution along the line 2.1 cm from the center of bilayer cloak. Clearly, the simulated distribution of electric field for the homogeneous dielectric medium (castor oil) is uniform, with the value of 6666 V/m. The presence of the steel layer tube and air layer tube causes the distortion of electric field, which can be confirmed by the position-dependent electric field. As seen in Fig. 4a, the electric field near the steel tube is strong and the direction of the electric field has a significant change. The maximum electric field can be determined to be 10,426 V/m. For the air shell tube, the electric field decreases near the shell to a minimum value of 4,661 V/m. For the bilayer coaxial cloak, the external field is almost undisturbed and one can obtain uniform electric field with the value about 6660 V/m. Thus, good cloak performance has been achieved.

In the measurement, an electrostatic instrument is used to quantify the corresponding electric field. The detailed information for electrostatic measuring instrument can be found in **Methods**, where the current readings in ampere meter are positive to the electrostatic field detected by the probe. Therefore, one can characterize the electrostatic field distribution by obtaining the corresponding current at a position. The measured results are presented in Fig. 4b, where the measured current distributions are in good agreement with the simulated electric field distributions, thus validating the feasibility of our scheme. It is noteworthy that the deviation can be attributed to the fabrication and measurement.

### Carpet electrostatic cloak

**Figure 5:**
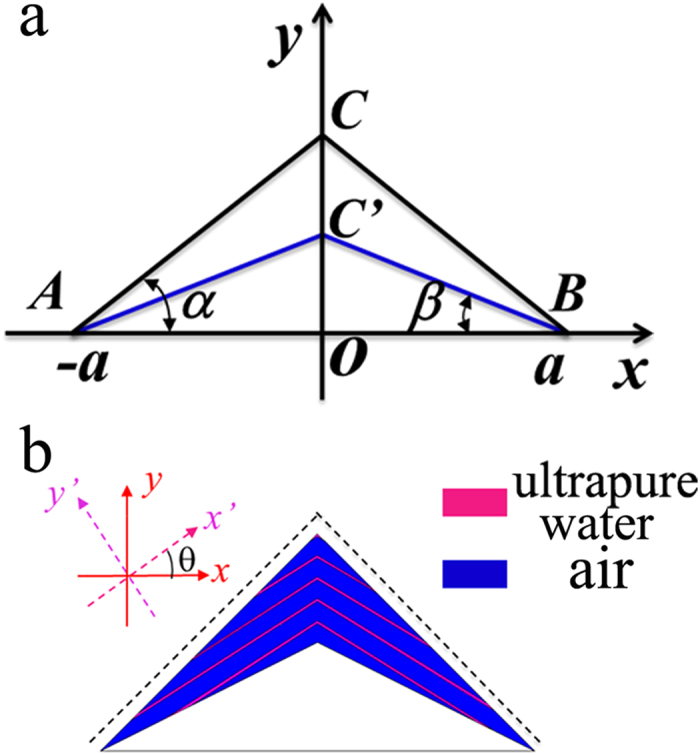
Design of carpet electrostatic invisibility cloak. (**a**) The transformation model for the carpet cloak. (**b**) The designed carpet cloak. The rotation angle between new and old coordinate systems is *θ*. The black dash lines represent the observation lines.

In addition to scattering cancelling method, the TO theory can also be used to obtain cloaking. As shown in Fig. 5a, the *x*–z PEC plane is connected to ground. In the transformation, the AOB is stretched to AC’B, while ACB remains unchanged. Thus, by placing the appropriate materials in the region AC’BCA, one can make the space of AC’BA invisible, then a carpet cloak is achieved. According to the theoretical analysis in **Methods**, the required components of dielectric constant tensor in the 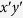 system are:


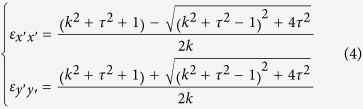


The rotation between the new and original coordinate system is


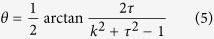


**Figure 6:**
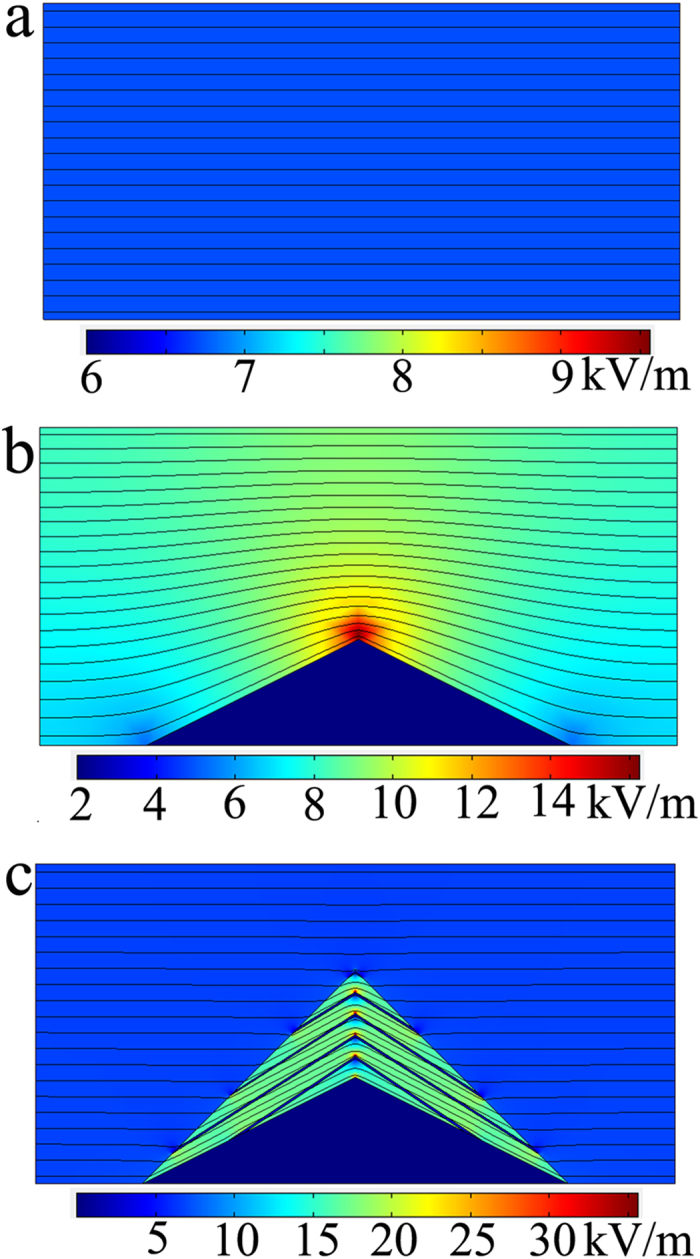
Numerical simulation for carpet cloak. The simulated electric field and isopotential lines: (**a**) background material (**b**) cone-shape PEC without cloak. (**c**) cone-shape PEC with carpet cloak.

**Figure 7:**
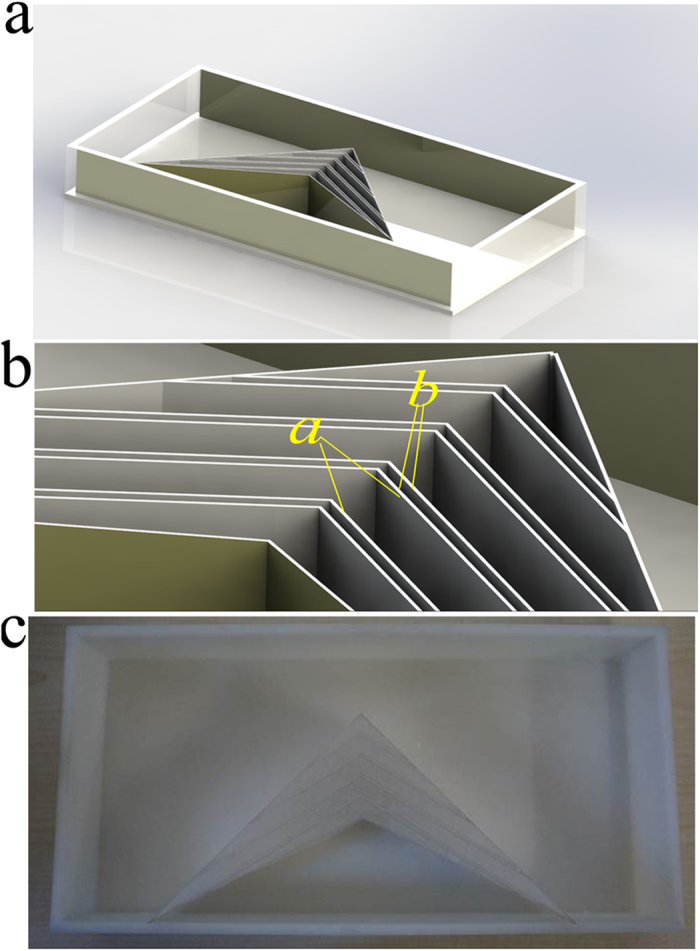
Experimental demonstration of carpet electrostatic field invisibility cloak. (**a**) The overall illustration of characterization of the device. (**b**) zoom, the geometric parameters for the carpet cloak are: *a* = 8.38 mm and *b* = 1.41 mm. (**c**) The photograph of fabricated sample.

**Figure 8:**
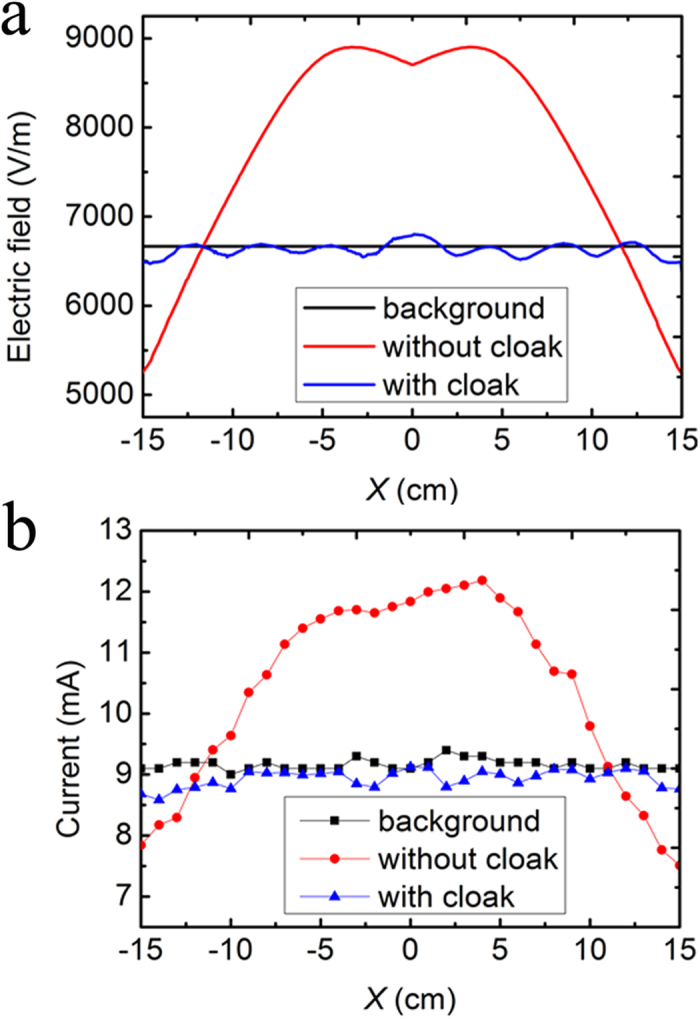
Characterization of carpet electrostatic field invisibility cloak. (**a**) Simulated electric field along the black dash line for the different cases. The black dash line can be seen in Fig. 5b. (**b**) The measured results of current in the electrostatic field measurement instrument for corresponding cases, respectively.

Here, 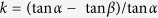, and 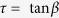. Clearly, the required material for the carpet cloak is homogenous but anisotropic. To achieve this anisotropy, one can use the metamaterial multilayer structure. Note that one component of the required dielectric constant is larger than background medium and the other one is smaller. Thus we employ air 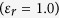 and ultrapure water 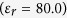 to fabricate such a metamaterial. In our study, the geometrical parameters for the carpet cloak are: AB = 2*a* = 20 cm, OC = *a* = 10 cm, OC’ = 0.5*a* = 5cm. As a result, one can obtain that: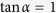, 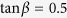. The designed metamaterial is given in Fig. 5b, where the filling ratio of the air is about 88%. Simulations are carried out to characterize the performance of the designed carpet cloak. In the simulations, −1000 V potential is biased between the two electrodes to generate nearly uniform electric field. The simulation results for the electric field and potential are shown in the Fig. 6a–c. Figure 6a shows the process how the uniform electric field is generated between the two electrodes, while Fig. 6b shows that the presence of isosceles triangular shaped PEC ridge causes serious distortion of the electric field and potential lines. The simulation results for the carpet cloak are provided in the Fig. 6c, where the distortion of the cloak disappears and only occurs in the carpet cloak, indicating good cloaking performance. As schematically shown in Fig. 7, the fabricated carpet cloak is a multilayer-groove structure, where the grooves are alternately filled with ultrapure water and air. The performance of the carpet cloak can also be evaluated by the electric field intensity along the dash lines as shown in Fig. 5b. The simulated electric field is presented in Fig. 8a. The electric field is uniform and has the value 6666 V/m. When the isosceles triangular shaped PEC ridge is placed in contact with the electrode, the electric field is strongly distorted. However, when the PEC is wrapped by carpet cloak, the distortion is cancelled and the electric field becomes uniform again. The measured results are shown in Fig. 8b, where the measured current shows good agreement with the simulated electric field, indicating the feasibility of our proposed scheme.

## Discussion

For EM wave propagation, the electric and magnetic fields couple to each other, causing great difficulties for the practical realization of invisibility cloaking in free space. The previous experimental works are usually classified into two categories. The first one is based on TO method, which however requires anisotropic, inhomogeneous, and even singular parameters for the magnetic and electric permittivity. Although a reduced scheme has been successfully proposed to obtain a cloak in free space, it is difficult to be extended to applications with high frequencies and three-dimensional configuration^3^. Another one is based on the scattering cancelling technology, which can avoid the problems of the TO-based cloak^34^. However, it is still imperfect, since only some scattering terms are cancelled. Thus, there is still a long way to go before a perfect cloak is obtained. However, as for electrostatic fields, realizing a perfect cloak (the cylindrical case for 2D or spherical one for 3D) in free space is easy. As demonstrated above, the perfect cloak can be achieved by directly solving the electrostatic equation. Using a bilayer structure, the cloak can be made with two kinds of naturally occurring materials. Although this bilayer cloak can only work for the two-dimensional case, it can be easily extended to three-dimensional ones. In addition, due to its simple configuration, it can be easily scalable. It’s worth mentioning that the bilayer cloak also has shortcoming: it only works for the inhomogeneous electric field, and the cloaking effect will be poor if a point source is used. This can be explained easily according to the previous work on static magnetic fields^12^ and thermal fields^21^, both of which show that the cloak performance under a pointlike diffuse source can be improved when the thickness of the bilayer cloak is reduced. The carpet invisibility cloak directly confirms the feasibility of the TO method for the electrostatic field. This powerful mathematical tool, with the combination of metamaterial, would provide a broad platform for the design of new devices. It is worth mentioning that the wavelength is infinite for the static case, which means that there is no subwavelength limits, thus the practical realization would be greatly simplified.

In summary, using cancelling technology and the transformation optics method, we demonstrate electrostatic field cloaks that can shield a specified region from the external field without any disturbance. These cloaks with homogeneous dielectric constants, can be readily obtained with naturally occurring materials. In addition, the simple structure can be easily extended to micro-nanoscale and three-dimensional configurations, thereby greatly enhancing practical realization and to enable applications like non-destructive detection. More importantly, our concept for manipulation of the electrostatic field can also be extended to other devices, such as, concentrators, rotators and illusion, which may find applications in various fields.

## Methods

### The theoretical analysis for bilayer cloak

**Figure 9:**
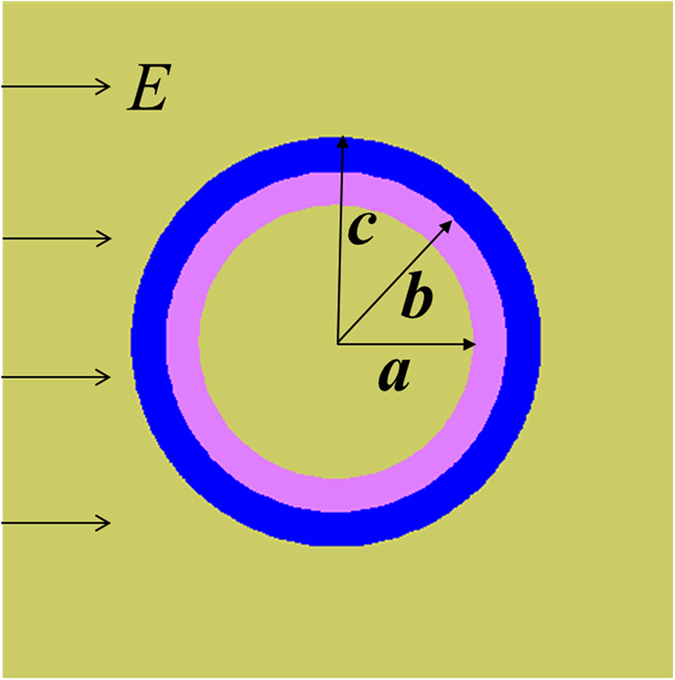
The corresponding physical model for bilayer cloak. The electric field is generated from high potential to low potential. The space is divided into three parts: *i* = 1 for interior (*r* < *a*), *i* = 2 for cloak shell (*a* < *r* < *b*) and *i* = 3 for exterior (*r* < *c*).

Figure 9 schematically illustrates the corresponding two-dimensional (2D) physical model of coaxial tubes where a uniform electric field 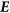 is produced from high potential to low potential. In the considered space, the electric potential is governed by the 2D Laplace’s equation 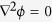, which can be expressed as


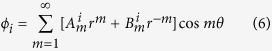


where 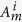 and 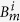 (*i* = 1, 2, 3) are constants to be determined, and 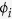 represents the potential in different regions: *i* = 1 for interior (*r* < *b*), *i* = 2 for the cloak cylindrical tube shell (*b* < *r* < *c*) and *i* = 3 for exterior (*r* > *c*). The dielectric constant for the background material, inner layer and outer layer of cloak shell tube are 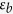, 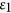 and 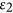, respectively.

Taking into account that 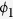 should be finite when 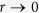, one can obtain that 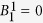. In addition, 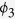 should tend to 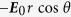 when 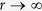, one only needs to consider 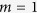. Furthermore, the electric potential and the normal component of electric field vector are continuous across the interfaces, one can obtain that


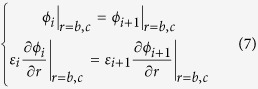


Here, 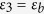, where 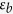 is the electric dielectric constant of the background. Combining the Equation 6 and 7, one can obtain


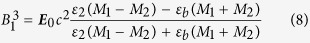


where 
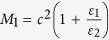
, 
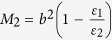
. By making 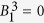, one can obtain


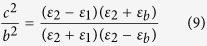


### The theoretical analysis for carpet cloak

In the transformation (see Fig. 5a), the AOB is stretched to AC’B, while ACB keeps unchanged. Thus, by placing the appropriate materials into the region AC’BCA, one can make the space of AC’BA invisible, thus a carpet cloak is achieved. According to the TO theory, one can obtain the required dielectric constant


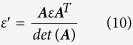


where 
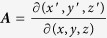
 is the Jacobian matrix. Here, the transformation equation is x´ = x


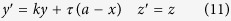


Then the required dielectric constant can be determined as


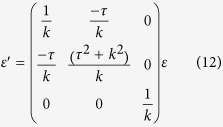


Here 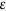 is the dielectric constant of the background medium. 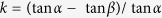, and 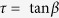. For 2D case, only in-plane parameters are considered and they form a symmetric 2 × 2 matrix. This matrix can be further diagonalized in the 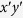 system, where the corresponding components of dielectric constant tensor are


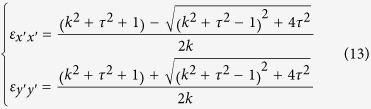


The rotation between the new and original coordinate system is


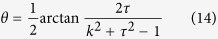


### Electric field measurement instrument

**Figure 10:**
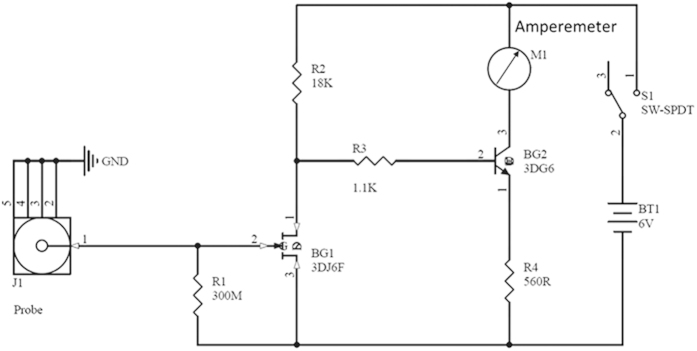
The schematic diagram for electrostatic measuring instrument. The current readings in ampere meter are positive to the electrostatic field detected by the probe.

The circuit of electrostatic field intensity measurement instrument is shown in Fig. 10. Electric field induction signal is detected using the field-effect tube, which has very high input resistance and is very sensitive to electric field induction around it. After switch K is thrown, the source of field-effect tube BG1 and the voltage between drains is lower when there is no electrostatic field around the probe of measurement instrument. There is no current getting through resistance R3, which cuts off BG2. Therefore, collector current of BG2 is zero, ampere meter is zero and the circuit is in the stationary state. When there is electrostatic field around the probe of measurement instrument, the charge begin to accumulate in probe because of electrostatic induction. The bias voltage produced between both ends of resistance R changes the internal resistance of BG1 source and the drain, which results in changes of the whole circuit state. There is current through resistance R3 after breaking over BG2 and the current amplified by BG2 is measured though ampere meter. The probe of measurement instrument can induct different quantity of electric charge in different position of electrostatic field because of different electrostatic field strength. Therefore, there is different collector current of BG2. The relative electrostatic field can be measured with this method.

## Additional Information

**How to cite this article**: Lan, C. *et al.* Electrostatic Field Invisibility Cloak. *Sci. Rep.* **5**, 16416; doi: 10.1038/srep16416 (2015).
